# Both Nuclear Size and DNA Amount Contribute to Midblastula Transition Timing in *Xenopus laevis*

**DOI:** 10.1038/s41598-017-08243-z

**Published:** 2017-08-11

**Authors:** Predrag Jevtić, Daniel L. Levy

**Affiliations:** Department of Molecular Biology, University of Wyoming, Laramie, WY 82071 USA

## Abstract

During early *Xenopus laevis* embryogenesis both nuclear and cell volumes decrease with the nuclear-to-cytoplasmic (N/C) volume ratio reaching a maximum at the midblastula transition (MBT). At the MBT, embryonic transcription is upregulated and cell cycles lengthen. Early studies demonstrated a role for the DNA-to-cytoplasmic ratio in the control of MBT timing. By altering nuclear size, we previously showed that the N/C volume ratio also contributes to proper MBT timing. Here we examine the relative contributions of nuclear size and DNA amount to MBT timing by simultaneously altering nuclear size and ploidy in *X. laevis* embryos. Compared to diploid embryos, haploids exhibited a delay in both zygotic gene expression and cell cycle lengthening, while diploid embryos with increased N/C volume ratios showed early expression of zygotic genes and premature lengthening of cell cycles. Interestingly, haploids with increased N/C volume ratios exhibited an intermediate effect on the timing of zygotic gene expression and cell cycle lengthening. Decreasing nuclear size in post-MBT haploid embryos caused a further delay in cell cycle lengthening and the expression of some zygotic genes. Our data suggest that both the N/C volume ratio and DNA amount contribute to the regulation of MBT timing with neither parameter being dominant.

## Introduction

Early *Xenopus laevis* embryogenesis is a powerful system to study mechanisms of developmental timing. After fertilization, the embryo undergoes twelve rapid synchronous cell divisions to reach the first major developmental landmark, the midblastula transition (MBT) (Nieuwkoop-Faber stage 8, cleavage 12) at ~7 hours post fertilization (hpf). At the MBT, cell cycles become longer and major zygotic transcription initiates^1–5^. At first a 1.2 mm single cell, the embryo is composed of several thousand 50 µm and smaller blastomeres by the MBT.

From stage 4 to stage 8, nuclear and cytoplasmic volumes decrease by 3-fold and ~70-fold, respectively. Consequently, the N/C volume ratio increases rapidly during early embryogenesis reaching a maximum at the MBT. We previously altered nuclear size in early *X. laevis* embryos and showed that this affected the timing of the onset of zygotic transcription and cell cycle lengthening^6^, implicating the N/C volume ratio in the control of MBT timing. A number of studies in different model organisms have shown that the DNA-to-cytoplasmic ratio is another mechanism that regulates MBT timing^1, 2, 7–15^. It has been proposed that the amount of genomic DNA present in an MBT embryo is sufficient to titrate maternally derived MBT inhibitors, thus leading to the onset of the MBT^1, 2^. Recently, putative limiting MBT inhibitors have been identified, including DNA replication initiation factors and histones^16–19^, and interestingly these factors do not seem to completely explain the altered gene expression and cell cycles associated with the MBT. Redundant mechanisms likely regulate this critical developmental transition^20, 21^, with nuclear volume potentially regulating the nuclear concentrations of DNA-binding MBT inhibitors.

To test the relative contributions of DNA amount and the N/C volume ratio to MBT timing, we simultaneously altered nuclear size and ploidy in early *X. laevis* embryos and examined the timing of zygotic transcription by *in situ* hybridization and qRT-PCR and the onset of longer cell cycles by bright-field time-lapse microscopy. As previously demonstrated, zygotic gene expression and cell cycle lengthening were delayed in haploid embryos and occurred prematurely in embryos with increased N/C volume ratios. Interestingly, we observed intermediate timing effects in haploid embryos with increased nuclear size while the MBT was further delayed in haploid embryos with reduced N/C volume ratios. These results suggest that both DNA amount and the N/C volume ratio contribute to proper MBT timing, with neither parameter having a dominant effect.

## Results and Discussion

**Figure 1:**
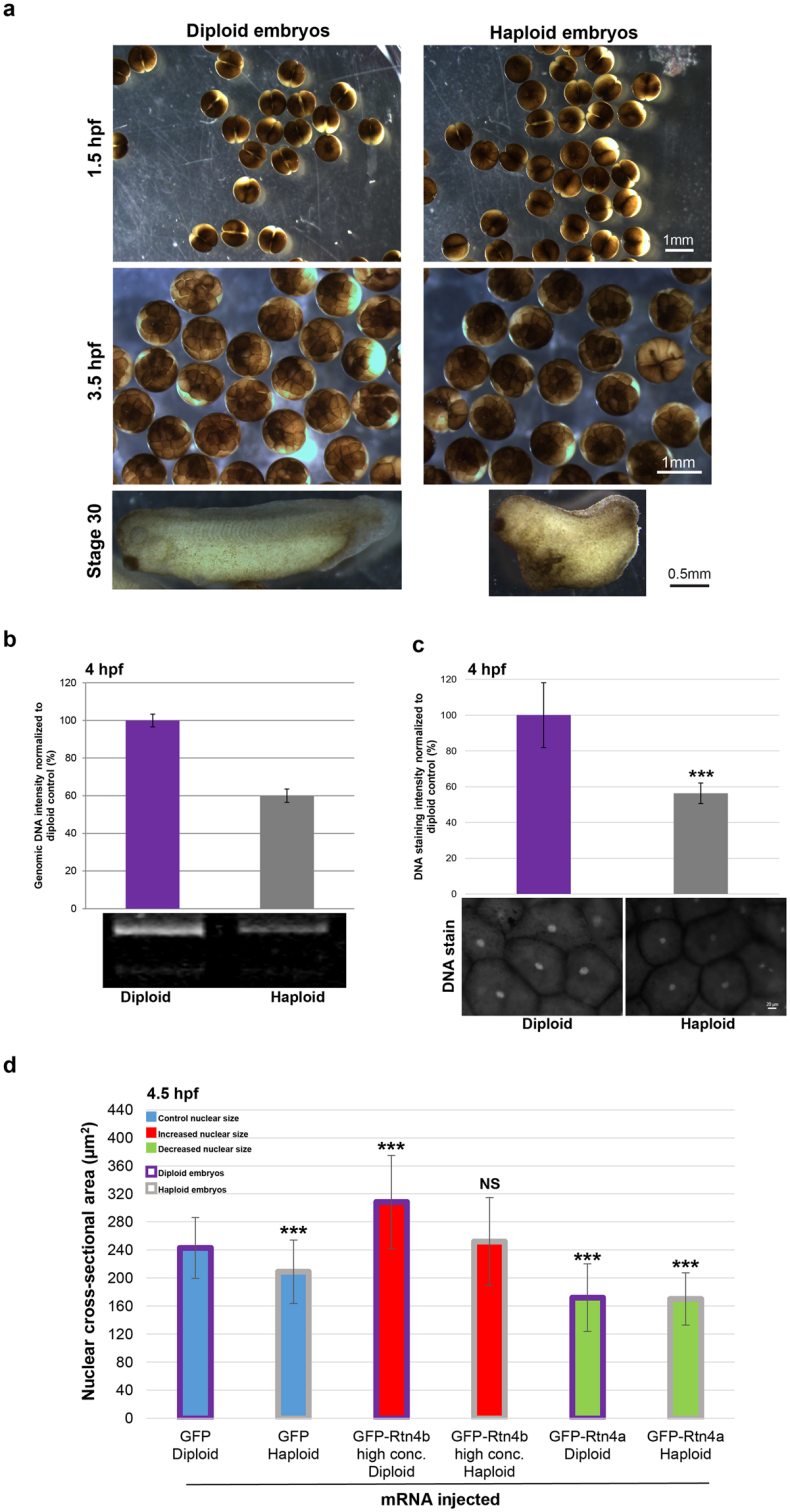
Altering ploidy in early *X. laevis* embryos **(a)** Haploid *X. laevis* embryos were generated by fertilizing eggs with UV-irradiated crushed *X. laevis* testes. Both haploid and diploid embryos underwent the first cleavage at ~1.5 hpf. Haploidy was verified by the irregular morphology of the tadpoles and their inability to develop past the swimming stage. **(b)** Genomic DNA was isolated from thirty 4 hpf embryos for each condition. 0.4 embryo equivalents of genomic DNA were run on 1.2% TAE agarose gels. DNA was visualized with ethidium bromide, quantified using ImageJ, and normalized to the diploid control. One representative gel and means from two independent experiments are shown. Error bars represent SD. **(c)** Whole un-arrested 4 hpf diploid and haploid embryos were fixed, bleached, stained for DNA with Sytox Green, and cleared. Animal pole cells were imaged for DNA intensity quantification. Several embryos from two different batches of diploid and haploid embryos were imaged. Total number of nuclei quantified: diploid, n = 38; haploid, n = 23. Error bars represent SD. ***p < 0.001. Scale bar, 20 µm. **(d)** Whole microinjected, un-arrested 4.5 hpf diploid and haploid embryos were fixed, bleached, stained for DNA with Sytox Green, and cleared. Animal pole cells were imaged and nuclear cross-sectional area quantified. We previously demonstrated that nuclear cross-sectional areas accurately reflect nuclear volumes^6^. Several embryos from two different batches of diploid and haploid embryos were imaged. Total number of nuclei quantified: GFP diploid, n = 55; GFP haploid, n = 47; GFP-Rtn4b high conc. diploid, n = 65; GFP-Rtn4b high conc. haploid, n = 49; GFP-Rtn4a diploid, n = 44; GFP-Rtn4a haploid, n = 95. The means from two independent experiments are shown. Error bars represent SD. ***p < 0.001; NS = not significant. All nuclear size differences between haploids were statistically significant by p < 0.001.

To generate haploid embryos, we fertilized *X. laevis* eggs with UV-irradiated *X. laevis* sperm. Haploid and diploid embryos underwent the first cleavage cell division at similar times. Haploidy was confirmed by quantification of total genomic DNA and DNA staining intensity in 4 hpf embryos, as well as by the altered morphology of stage 30 haploid embryos (Fig. 1a–c). One-cell *X. laevis* haploid and diploid embryos generated from the same batch of eggs were microinjected with different mRNAs expressing nuclear scaling factors to increase or decrease nuclear size. In particular, microinjecting high concentrations of GFP-Rtn4b mRNA increased nuclear size while GFP-Rtn4a mRNA decreased nuclear size. Microinjection of GFP mRNA served as a control^6^. Compared to diploid control embryos, haploid controls exhibited ~10% smaller nuclear cross-sectional areas, as previously reported^22^. Nuclear size was also altered in microinjected haploid embryos, with ~20% increased or decreased nuclear areas compared to haploid controls (Fig. 1d).

**Figure 2:**
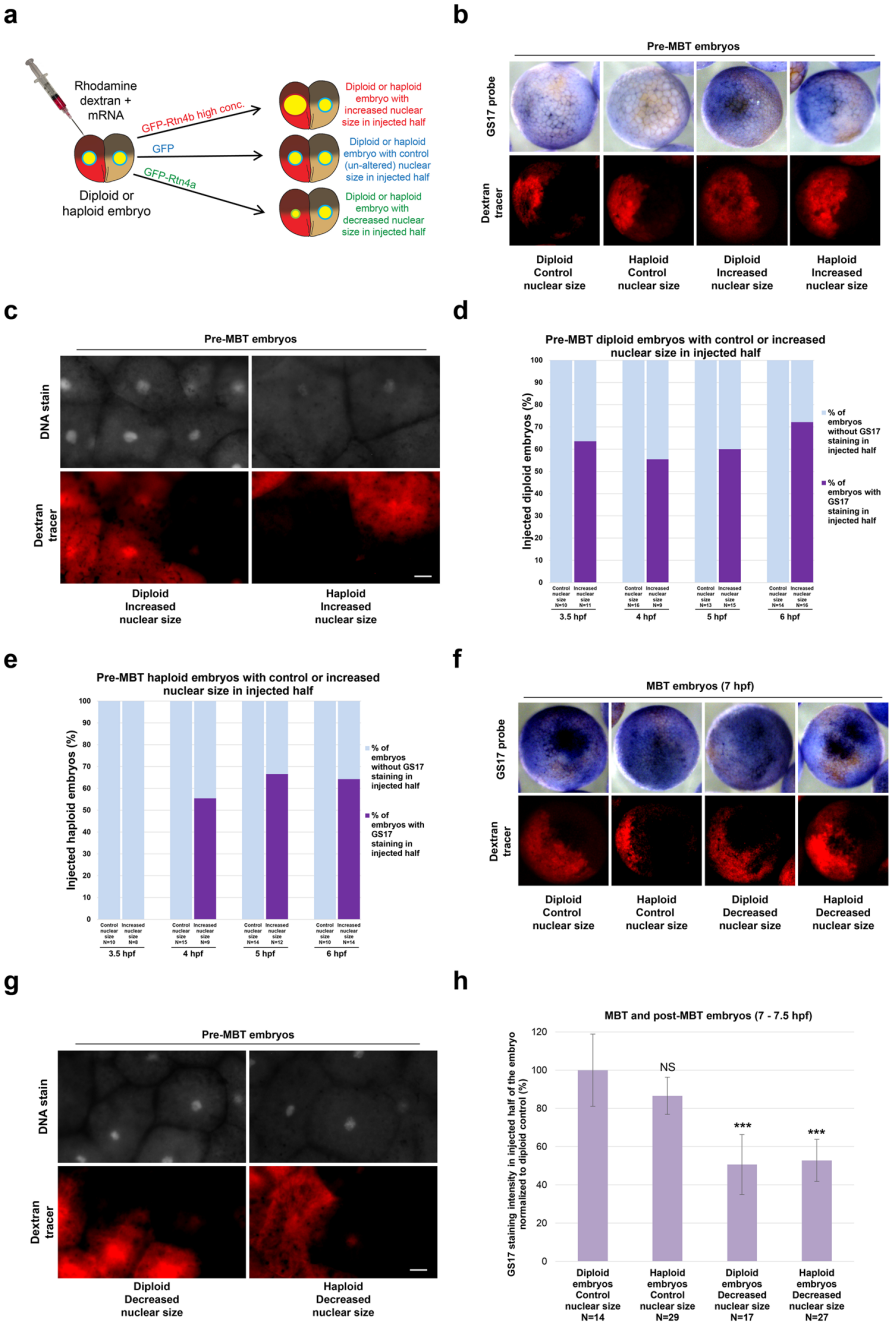
GS17 *in situ* hybridization in embryos with altered nuclear size and ploidy **(a)** One blastomere of a two-cell haploid or diploid embryo was coinjected with rhodamine-labeled dextran as a tracer and mRNA to alter nuclear size in half of the embryo. For control embryos, 3.5, 4, 5, 6, and 7 hpf correlate to stage 6, 6.5, 7, early 8, and 8, respectively. **(b)** GS17 *in situ* hybridization was performed on pre-MBT embryos with increased nuclear size in half of the embryo. Bright-field images of embryos stained for GS17 (purple) are shown in the top panels. The corresponding rhodamine (red) fluorescence images indicating cells in the embryo that received the microinjected mRNA are shown in the bottom panels. Representative embryos are shown. **(c)** 5 hpf diploid and haploid embryos with increased nuclear size in the injected halves were stained with Sytox Green and cleared for imaging. The top panels are fluorescent images of embryos stained for DNA. The bottom panels are the corresponding rhodamine fluorescence images indicating cells in the embryo that received the microinjected mRNA. Representative embryos are shown. Scale bar, 20 µm. **(d,e)** The graphs show the percentage of pre-MBT diploid **(d)** and haploid **(e)** embryos with (dark purple) or without (light blue) differential GS17 staining. Embryos were scored as showing differential GS17 staining as long as some cells that received the microinjected mRNA stained positively for GS17. N = number of embryos. **(f)** 7 hpf embryos with decreased nuclear size in half of the embryo were subjected to GS17 *in situ* hybridization. Imaging was performed as in **(b)**. **(g)** 5 hpf diploid and haploid embryos with decreased nuclear size in the injected halves were stained with Sytox Green and cleared. Imaging was performed as in **(c)**. Scale bar, 20 µm. **(h)** The graph shows GS17 staining intensity in injected halves of 7-7.5 hpf diploid and haploid embryos with control or decreased nuclear size, normalized to the diploid control. N = number of embryos. Error bars represent SD. ***p < 0.001; NS = not significant.

To test if altering nuclear size in early stage haploid embryos affects the onset of zygotic gene expression, we performed whole-mount *in situ* hybridization to detect GS17, a transcript expressed at high levels at the MBT^23, 24^. We microinjected one blastomere at the two-cell stage to alter nuclear size in half of the embryo (Fig. 2a,c,g) and used differential GS17 staining in the embryo as an indicator of altered MBT timing^6^. Diploid and haploid embryos microinjected with control GFP mRNA showed no pre-MBT GS17 staining (Fig. 2b,d,e), and by 7 hpf both halves of these embryos were positive for GS17 staining (Fig. 2f). Quantification of GS17 staining intensity in whole 7 hpf control embryos showed less intense GS17 staining in haploid embryos compared to diploid embryos, consistent with an expected delay in zygotic gene expression in haploids (Fig. S1a).

Both diploid and haploid pre-MBT embryos with increased nuclear size exhibited premature expression of GS17 (Fig. 2b–e), with less intense GS17 staining observed in haploid embryos with increased nuclear size compared to diploid embryos with increased nuclear size (Fig. S1b). While differential GS17 staining was already observed in 3.5 hpf diploid embryos, haploid embryos did not exhibit differential staining until 4 hpf. We note that we never observed 100% of embryos with differential GS17 staining and that not all cells in injected halves stained for GS17 (Fig. 2d,e), as previously reported and discussed^6^. We quantified nuclear sizes on a cell-by-cell basis in 4.5 hpf haploid embryos that had been microinjected to increase nuclear size. We observed that GS17-positive cells exhibited larger nuclear sizes than GS17-negative cells (Fig. S1c), so increased nuclear size correlates with GS17 expression in haploids. These results indicate that an increased N/C volume ratio correlates with premature zygotic gene expression in both haploid and diploid embryos, with an accelerated MBT onset in diploids relative to haploids. Consistent with nuclear size contributing to MBT timing in both haploids and diploids, we observed weaker GS17 staining in haploid and diploid embryos when we decreased nuclear size, suggestive of a delay in MBT timing (Fig. 2f–h).

We next assessed how ploidy affects MBT timing in cells with similar N/C volume ratios. We identified GS17-positive cells in 4.5 hpf haploid and diploid embryos that had been microinjected to increase nuclear size in which nuclear sizes were comparable. The haploid cells exhibited weaker GS17 staining, in spite of having similar nuclear sizes to the diploids (Fig. S1d). Furthermore, 7 hpf haploid cells with increased nuclear size showed less intense GS17 staining than control diploid cells with similarly sized nuclei (Fig. S1e), consistent with ploidy influencing the timing of zygotic transcription onset in addition to nuclear size.

**Figure 3:**
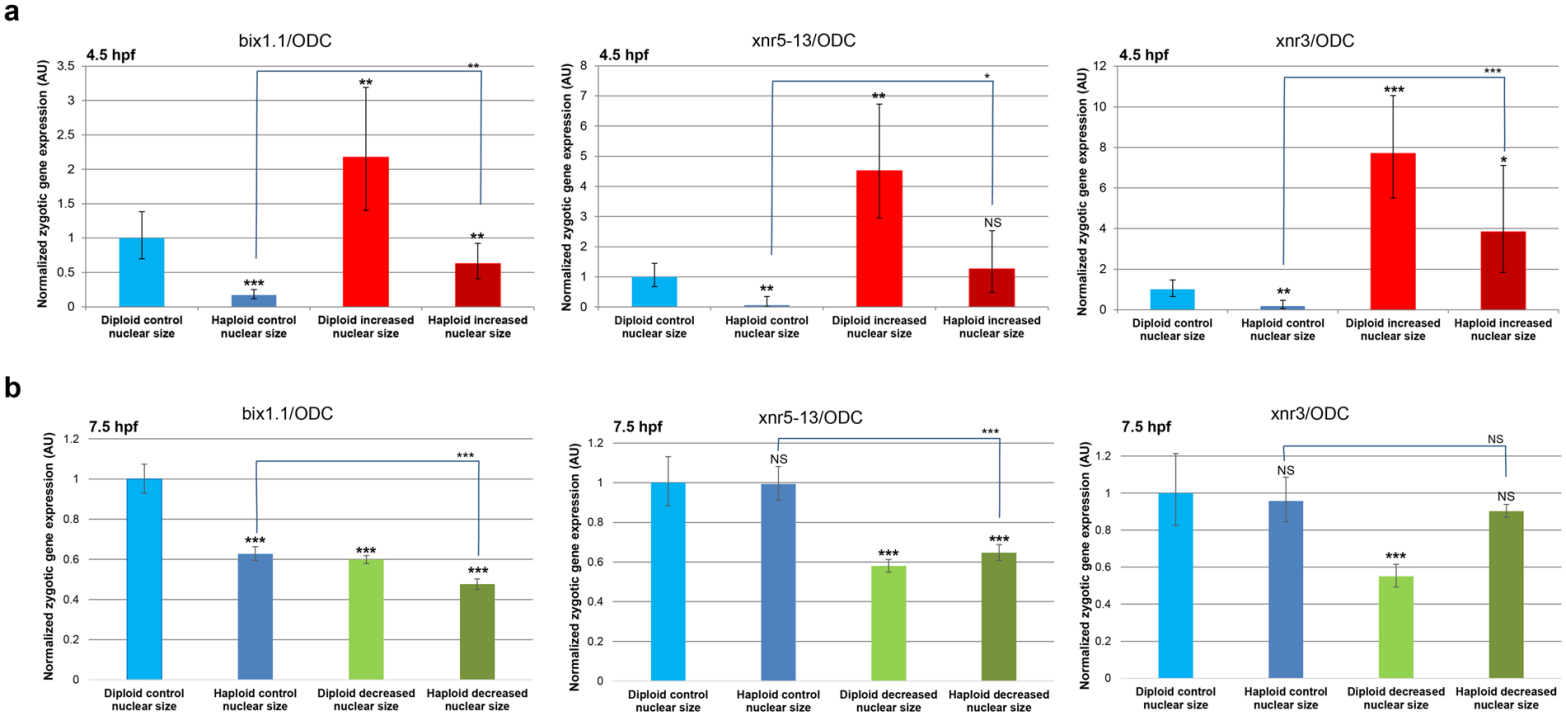
Quantification of zygotic transcripts by qRT-PCR in embryos with altered nuclear size and ploidy. One-cell diploid and haploid embryos were microinjected with mRNAs to increase **(a)** or decrease **(b)** nuclear size and allowed to develop to 4.5 hpf **(a)** or 7.5 hpf **(b)**. Total RNA was isolated from 30 embryos for each condition and converted to cDNA. Expression levels of three zygotic genes (xnr5-13, xnr3, and bix1.1) were determined by qRT-PCR, normalized to ODC. Gene-expression levels are plotted in arbitrary units (AU) relative to control diploid embryos. The means from two independent experiments are shown. Error bars represent SD. ***p < 0.001; **p < 0.01; *p < 0.05; NS = not significant.

To examine a greater number of MBT transcripts and more precisely quantify transcript levels, we performed qRT-PCR on 4.5 hpf haploid and diploid embryos with increased nuclear size to quantify relative transcript levels^4, 25^. While this approach does not directly determine the timing of zygotic gene expression, differences in expression levels suggest differences in the timing of expression onset for a given gene. As expected, 4.5 hpf diploid embryos with increased nuclear size expressed up to 7-fold higher levels of three additional MBT transcripts (bix1.1, xnr5–13, xnr3) compared to control diploid embryos^6^ (Fig. 3a). Conversely, expression of these MBT transcripts was reduced in control haploid embryos relative to control diploids, consistent with a reduced DNA-to-cytoplasm ratio delaying MBT onset^2^ (Fig. 3a). Strikingly, 4.5 hpf haploid embryos with increased nuclear size showed increased expression of MBT transcripts compared to control haploid embryos, consistent with the N/C volume ratio still affecting MBT timing even when ploidy is reduced (Fig. 3a). In haploids with increased nuclear size, transcript expression levels were intermediate between control haploids and diploids with increased nuclear size, approaching the expression levels observed in control diploids (Fig. 3a).

In haploid post-MBT embryos, expression of MBT transcripts was either reduced or similar to diploid control embryos (Fig. 3b). Decreasing nuclear size in diploids led to reduced expression of all three MBT transcripts compared to diploid controls, as previously reported^6^. On the other hand, haploids with reduced nuclear size exhibited variable effects on MBT transcript levels, with expression similar to haploid controls (xnr3), similar to diploids with decreased nuclear size (xnr5-13), or less than diploids with decreased nuclear size (bix1.1) (Fig. 3b). In post-MBT embryos, perhaps the zygotic transcription response to ploidy and nuclear size is transcript-dependent. Taken together, these data suggest that both the N/C volume ratio and DNA-to-cytoplasm ratio contribute to proper MBT timing and that neither parameter is dominant over the other.

**Figure 4:**
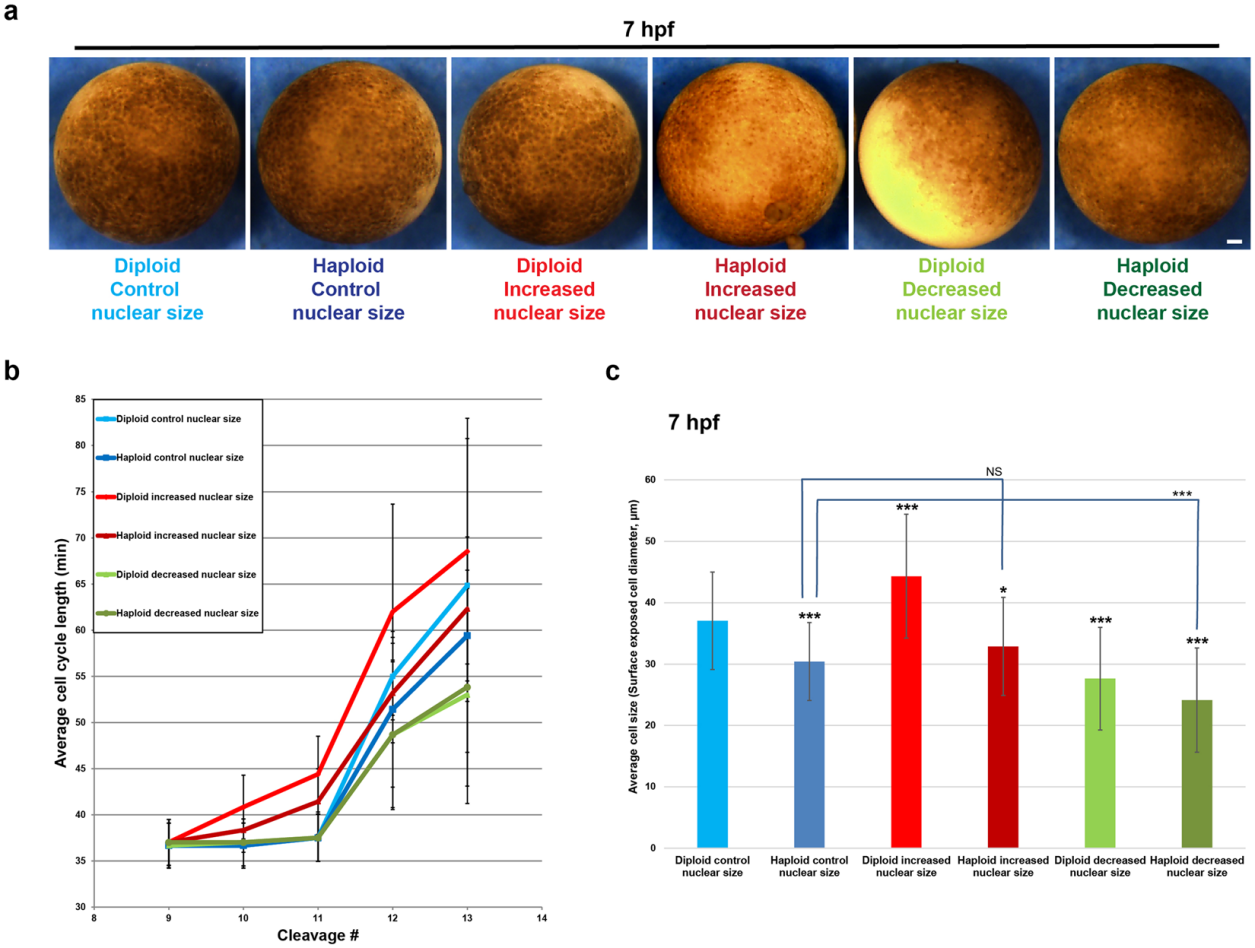
Cell cycle timing in embryos with altered nuclear size and ploidy. One-cell diploid and haploid embryos were microinjected with mRNA to alter nuclear size, and bright-field time-lapse imaging was performed on the animal pole surface at 5-min intervals at 21 °C. **(a)** Still frame images of 7 hpf embryos. Scale bar, 100 µm. **(b)** Cell-cycle lengths were measured for at least five cells per embryo starting at the ninth cell division for three embryos per condition. Data from two independent experiments are shown; error bars represent SD. **(c)** Cell sizes were estimated by quantifying the diameter of surface-exposed cells on the animal pole of 7 hpf embryos. For each embryo, several random regions were selected for cell size quantification. Cells from at least two embryos were quantified for each condition. Total number of cells quantified: diploid control, n = 34; haploid control, n = 34; diploid increased nuclear size, n = 42; haploid increased nuclear size, n = 43; diploid decreased nuclear size, n = 22; haploid decreased nuclear size, n = 36. Error bars represent SD. ***p < 0.001; *p < 0.05; NS = not significant.

Next, we analyzed time-lapse movies of microinjected diploid and haploid embryos to determine at which cleavage division number the cell cycle timing became longer (Figs. 4a and S2). Generally, control embryos cleaved synchronously and rapidly until the 12^th^ cleavage, when cell cycles lengthened in both diploid and haploid embryos, but to a slightly greater extent in diploids (Fig. 4b). Decreasing nuclear size correlated with reduced average cell cycle lengths at the 12^th^ cleavage in both diploid and haploid embryos compared to controls. Conversely, increasing nuclear size in diploid embryos was associated with premature cell cycle lengthening after only the 10^th^ cleavage, as expected^6^. Interestingly, haploid embryos with increased nuclear size also exhibited premature lengthening of cell cycles but with cell cycle times intermediate between control haploids and diploids with increased nuclear size (Fig. 4b). These findings were supported by cell size measurements that serve as a proxy for the number of cell divisions (Fig. 4a and c). These data suggest that both DNA amount and nuclear size influence the timing of the onset of cell cycle lengthening associated with the MBT.

We have shown that both haploid and diploid embryos with increased nuclear size express higher levels of zygotic transcripts and exhibit early onset of longer cell cycles compared to their control haploid and diploid counterparts. Comparing embryos with increased N/C volume ratios, MBT onset in haploids was still delayed relative to diploids. Thus, our results suggest that both the N/C volume ratio and DNA-to-cytoplasm ratio contribute to the proper regulation of MBT timing, with neither parameter having a dominant effect over the other.

How might both nuclear size and DNA amount influence MBT timing? While ploidy affects nuclear size (Fig. 1d), we have shown that when comparing haploid and diploid cells with similarly sized nuclei, GS17 staining is weaker in the haploid (Fig. S1d–e). These results support the idea that there are ploidy effects on MBT timing that are independent of nuclear size, consistent with models invoking recently identified regulators of MBT onset, including DNA replication initiation factors, their regulatory subunits, and histones^16, 17, 19, 26^. While increasing amounts of DNA in the embryo are proposed to titrate these maternally-derived factors to determine MBT timing, nuclear volume might play a role in this process by regulating the nuclear concentrations of these factors. It is also possible that different hallmarks of the MBT are controlled through different mechanisms or limiting components. For example, we observed transcript-specific differences in the response of post-MBT embryos to altered ploidy and decreased nuclear size (Fig. 3b). Perhaps some zygotic genes preferentially respond to changes in the N/C volume ratio while others respond to total DNA amount. The transcriptional response to both ploidy and increased nuclear size was stronger in 4.5 hpf embryos (Fig. 3a) than at 7.5 hpf (data not shown), consistent with ploidy and decreased nuclear size eliciting relatively moderate transcriptional effects in 7.5 hpf embryos (Fig. 3b) and suggesting that the N/C volume ratio and ploidy play key timing roles before the MBT as opposed to at the MBT. The results presented here will help to inform future mechanistic studies into the timing of this important developmental transition.

## Methods

### Plasmids and cloning

The coding sequence of human Rtn4b (DNASU Plasmid Repository Clone ID HsCD00081743) was cloned into pCS107-GFP3STOP (obtained from John Wallingford) generating an N-terminal GFP fusion to Rtn4b (pDL34). An SP6 promoter was cloned upstream of the human Rtn4a coding sequence in plasmid hRtn4a-GFP pAcGFP-N1 (obtained from Gia Voeltz) to generate new plasmid pDL49. For control injections, we used GFP mRNA expressed from pCS107-GFP3STOP.

### *Xenopus laevis* embryos and microinjections

*X. laevis* embryos were obtained by *in vitro* fertilization of freshly laid *X. laevis* eggs with crushed *X. laevis* testes^27^. To generate haploid *X. laevis* embryos, a similar protocol was used except that fresh *X. laevis* testes were crushed in 1x MMR (20x MMR = 2 mM EDTA, 2 M NaCl, 40 mM KCl, 20 mM MgCl_2_, 40 mM CaCl_2_, 100 mM HEPES pH 7.8) and irradiated twice with 30,000 J/cm^2^ ultraviolet radiation using a Stratalinker (Stratagene)^22^. Only batches with greater than 90% fertilization efficiency were used. Twenty minutes after fertilization, embryos were de-jellied in 2.5% cysteine pH 7.8 dissolved in 1/3x MMR. Embryos were staged according to^5^. All *Xenopus* procedures and studies were conducted in accordance with the US Department of Health and Human Services Guide for the Care and Use of Laboratory Animals. Protocols were approved by the University of Wyoming Institutional Animal Care and Use Committee (Assurance # A-3216-01).

Following linearization of pCS107-GFP3STOP, pDL34, and pDL49, mRNA was expressed from the SP6 promoter using the mMessage mMachine kit (Ambion). Embryos at the one-cell or two-cell stage were transferred to 1/3 MMR plus 2.5% Ficoll and injected with 10 nL volumes using a PicoSpritzer III (Parker). After 45 minutes, the buffer was changed to 1/3x MMR and embryos were allowed to develop to desired stages.

### Whole-mount *in situ* hybridization and microscopy

One blastomere of a two-cell embryo was co-microinjected with mRNA and 50 ng lysine-fixable tetramethylrhodamine-labeled dextran, 70000 MW (Invitrogen, D1818) as a fluorescent marker for the injected half. For control experiments, mRNA expressing GFP was used. At different stages, embryos were fixed with MEMFA consisting of 1 part 10x MEMFA salts (1 M MOPS, 20 mM EGTA, 10 mM MgSO_4_), 1 part 37% formaldehyde, and 8 parts water for 2 hours and stored in ethanol at −20 °C. Embryos were rehydrated in a methanol gradient, washed in PBST, and permeabilized with 10 µg/ml proteinase K for 7 minutes. Embryos were washed in 0.1 M triethanolamine 2 × 5 minutes, then 2 × 5 minutes in 0.1 M triethanolamine supplemented with acetic anhydride (Sigma) (12.5 µl acetic anhydride to 5 ml 0.1 M triethanolamine). Embryos were washed in PBST and fixed for 20 minutes in 4% paraformaldehyde in PBST. Next, embryos were incubated for 5 hours at 60 °C in hybridization buffer (50% formamide, 5x SSC, 1 mg/ml Torula RNA, 100 µg/mL heparin, 1x Denhart’s, 0.1% Tween 20, 0.1% CHAPS, 10 mM EDTA). The digoxigenin-labeled anti-GS17 probe was added at 1 µg/ml (see below for preparation) and overnight hybridization was performed at 60 °C. Embryos were washed 2 × 3 minutes in 2x SSC at 60 °C, 3 × 20 minutes in 2x SSC at 60 °C, and then 30 minutes at 37 °C in 2x SSC supplemented with 20 µg/ml RNase A and 10 µg/ml RNase T_1_. Embryos were washed twice in 0.2x SSC for 30 minutes at 60 °C and 2 × 7.5 min at room temperature in 1x MAB (100 mM maleic acid, 150 mM NaCl), before being blocked with MAB + 2% BMB (Boehringer Mannheim Blocking Reagent) for 2 hours at room temperature. Embryos were then incubated with anti-digoxigenin alkaline phosphatase tagged antibodies (Sigma) diluted 1:3000 in MAB + 2% BMB for 4 hours at room temperature, and washed overnight at 4 °C in MAB. Alkaline phosphatase detection was performed with NBT/BCIP consisting of 4.5 mg/ml NBT (Sigma) and 3.5 mg/ml BCIP (Sigma) in alkaline phosphatase buffer (100 mM Tris pH 9.5, 50 mM MgCl_2_, 100 mM NaCl, 0.1% Tween 20, 2 mM levamisol). After staining, embryos were fixed overnight at room temperature in MEMFA and bleached in bleaching solution (1% H_2_0_2_, 5% formamide, 0.5x SSC) under direct light for 1–2 hours at room temperature. Images were acquired with an Olympus SZX16 research fluorescence stereomicroscope equipped with Olympus DP72 camera, 11.5x zoom microscope body, and SDFPLAPO1XPF objective.

To generate the anti-GS17 probe, we obtained a plasmid containing the full-length cDNA sequence of *X. laevis* GS17 in pCMV-SPORT6 (Open Biosystems, MXL1736-99234694). The plasmid was linearized and anti-sense probe was transcribed *in vitro* from the T7 promoter (NEB), with digoxigenin-11-UTP (Roche) included in the reaction. The probe was precipitated, washed, resuspended in hybridization buffer at 10 µg/ml, and stored at −80 °C. This protocol was adapted from^27^.

GS17 staining intensity was quantified using Metamorph software (Molecular Devices) from original images acquired using the same exposure time. For Figs 2h, S1b,d and e, the average GS17 staining intensity in the injected halves of embryos was measured within a small circular region and averaged. For Fig. S1a, average GS17 staining intensity of whole 7 hpf control diploid and haploid embryos was quantified and averaged. GS17 staining intensity measurements were corrected for background but not ploidy.

### Nuclear size and DNA staining intensity quantification

Embryos previously stained by *in situ* hybridization were washed 3 × 1 hour at room temperature in TBST (TBS + 0.1% Tween 20), incubated overnight at 4 °C with Sytox Green nucleic acid stain (Life Technologies, S7020, 1:1000 dilution in TBST), and washed 3 × 1 hour in TBST at room temperature. Embryos were dehydrated in methanol, cleared in 2:1 benzyl benzoate:benzyl alcohol, and imaged in circular ~1.5 mm deep chambers covered with 25 × 25 mm glass coverslips. Images of nuclei were acquired using the same exposure time with an Olympus BX51 fluorescence microscope, Olympus UPLFLN 20x (N.A. 0.50, air) objective, and QIClick Digital CCD Camera (model QIClick-F-M-12, mono, 12-bit). DNA staining intensity and nuclear cross-sectional area were quantified from original thresholded images using cellSens Dimension imaging software (Olympus).

### Quantitative real-time PCR (qRT-PCR)

For each condition, 30 embryos were lyzed and total RNA was purified from 3 embryo equivalents using the Absolutely RNA Microprep Kit (Agilent Technologies). First-strand cDNA synthesis was performed with random primers using the AffinityScript Multiple Temperature cDNA Synthesis Kit (Agilent technologies). qPCR was performed using FastStart Essential DNA Green Master Mix (Roche) and a Roche LightCycler 96. PCR efficiency was verified for each primer set, and only primer pairs having amplification efficiencies within 10% of perfect were used. Each 15 µl reaction contained 10 ng cDNA and 0.5 µM of each primer. All reactions were performed in quadruplicate, at a minimum. The following program was used: 95 °C for 600 seconds; 45 cycles of 95 °C for 10 seconds, 60 °C for 15 seconds, 72 °C for 15 seconds; 95 °C for 10 seconds; 65 °C for 60 seconds; increasing at 0.2 °C/second from 65 °C to 97 °C. Gene expression was normalized to ODC (*Ornithine decarboxylase*) and calculated by the DDCt method. Transcript levels were not normalized for ploidy. Primer sequences were (5′ to 3′):

BIX1.1FW AGCACCTACTTCTCCTCCAGT

BIX1.1REV GCTTGCTGTACTGGACTCTGT

XNR3FW CGATGCCTCCAGTCCTACAG

XNR3REV TCCTTGAAATTCTCTGGCTCCA

XNR5-13FW CCTTTCACTAGGGCATGGGA

XNR5-13REV GGTGAAGGTTCCAGTCTGTGT

ODCFW CTGGAGGAAGGCTTCTCTGC

ODCREV TGTCGCCAAGATCAGCAACA

### Isolation of genomic DNA

For each condition, 30 embryos were homogenized in 900 µl of DNA extraction buffer (10 mM Tris pH 8.0, 0.2 mM EDTA, 50 µg/ml RNase A, 0.5% SDS) and incubated at 37 °C for 1 hour. Proteinase K was added at 100 µg/ml and samples were incubated at 50 °C for 2 hours. Samples were extracted in one volume of phenol:chloroform:isoamyl alcohol (25:24:1). Genomic DNA was ethanol precipitated and resuspended in 60 µl of 10 mM Tris pH 8.5. This protocol was adapted from^28^. 0.4 embryo equivalents of genomic DNA were run on 1.2% TAE agarose gels and visualized by staining with ethidium bromide. DNA amount was quantified using ImageJ.

### Bright-field time-lapse imaging of whole embryos

One-cell embryos were microinjected as already described and allowed to develop at room temperature. Time-lapse imaging was performed with an Olympus stereomicroscope (Olympus SZX16 research fluorescence stereomicroscope equipped with Olympus DP72 camera, 11.5× zoom microscope body, and SDFPLAPO1XPF objective) at room temperature on the animal pole starting at 4 hpf. Images were acquired every 5 minutes. Discontinuous light was used to illuminate embryos, controlled with a digital adjustable cycle timer (CT-1 Short Cycle Timer, Innovative Grower Corp). The number of time intervals between cell divisions was counted to determine cell cycle lengths. Division timing was measured for at least 5 cells per embryo and for each cleavage. The diameter of surface-exposed cells was measured using cellSens Dimension imaging software (Olympus).

### Statistics

Averaging and statistical analysis were performed for independently repeated experiments. Two-tailed Student’s t-tests assuming equal variances were performed in Excel (Microsoft) to evaluate statistical significance. The p-values, number of independent experiments, and error bars are denoted in the Figure Legends.

### Data availability

All data generated or analyzed during this study are available from the corresponding author on reasonable request.

## Electronic supplementary material

Supplementary Information

## References

[1] Newport J, Kirschner M (1982). A major developmental transition in early Xenopus embryos: I. characterization and timing of cellular changes at the midblastula stage. Cell.

[2] Newport J, Kirschner M (1982). A major developmental transition in early Xenopus embryos: II. Control of the onset of transcription. Cell.

[3] Newport JW, Kirschner MW (1984). Regulation of the cell cycle during early Xenopus development. Cell.

[4] Collart C (2014). High-resolution analysis of gene activity during the Xenopus mid-blastula transition. Development.

[5] Nieuwkoop, P. D. & Faber, J. *Normal Table of Xenopus laevis (Daudin)*. 2nd edn, (North-Holland Publishing Company, 1967).

[6] Jevtic P, Levy DL (2015). Nuclear size scaling during Xenopus early development contributes to midblastula transition timing. Curr Biol.

[7] Clute P, Masui Y (1995). Regulation of the appearance of division asynchrony and microtubule-dependent chromosome cycles in Xenopus laevis embryos. Dev Biol.

[8] Clute P, Masui Y (1997). Microtubule dependence of chromosome cycles in Xenopus laevis blastomeres under the influence of a DNA synthesis inhibitor, aphidicolin. Dev Biol.

[9] Kobayakawa Y, Kubota HY (1981). Temporal pattern of cleavage and the onset of gastrulation in amphibian embryos developed from eggs with the reduced cytoplasm. J Embryol Exp Morphol.

[10] Edgar BA, Kiehle CP, Schubiger G (1986). Cell cycle control by the nucleo-cytoplasmic ratio in early Drosophila development. Cell.

[11] Edgar BA, Schubiger G (1986). Parameters controlling transcriptional activation during early Drosophila development. Cell.

[12] Kane DA, Kimmel CB (1993). The zebrafish midblastula transition. Development.

[13] Signoret J, Lefresne J (1973). Contribution a l’etude de la segmentation de l’oeuf d’axolotl. Annales d’Embryologie et de Morphogenese.

[14] Mita I (1983). Studies on Factors Affecting the Timing of Early Morphogenetic Events During Starfish Embryogenesis. The Journal of Experimental Zoology.

[15] Mita I, Obata C (1984). Timing of Early Morphogenetic Events in Tetraploid Starfish Embryos. The Journal of Experimental Zoology.

[16] Collart C, Allen GE, Bradshaw CR, Smith JC, Zegerman P (2013). Titration of four replication factors is essential for the Xenopus laevis midblastula transition. Science.

[17] Murphy CM, Michael WM (2013). Control of DNA replication by the nucleus/cytoplasm ratio in Xenopus. J Biol Chem.

[18] Vastag L (2011). Remodeling of the metabolome during early frog development. PLoS One.

[19] Amodeo AA, Jukam D, Straight AF, Skotheim JM (2015). Histone titration against the genome sets the DNA-to-cytoplasm threshold for the Xenopus midblastula transition. Proc Natl Acad Sci USA.

[20] Veenstra GJ (2002). Early Embryonic Gene Transcription in Xenopus. Advances in Developmental Biology and Biochemistry.

[21] Almouzni G, Wolffe AP (1995). Constraints on transcriptional activator function contribute to transcriptional quiescence during early Xenopus embryogenesis. EMBO J.

[22] Levy DL, Heald R (2010). Nuclear size is regulated by importin alpha and Ntf2 in Xenopus. Cell.

[23] Krieg PA, Melton DA (1985). Developmental regulation of a gastrula-specific gene injected into fertilized Xenopus eggs. EMBO J.

[24] Harvey RP, Tabin CJ, Melton DA (1986). Embryonic expression and nuclear localization of Xenopus homeobox (Xhox) gene products. EMBO J.

[25] Yanai I, Peshkin L, Jorgensen P, Kirschner MW (2011). Mapping gene expression in two Xenopus species: evolutionary constraints and developmental flexibility. Dev Cell.

[26] Joseph, S. R. *et al*. Competition between histone and transcription factor binding regulates the onset of transcription in zebrafish embryos. *Elife* **6**, doi:10.7554/eLife.23326 (2017). 10.7554/eLife.23326 PMC5451213 28425915

[27] Sive, H. L., Grainger, R. M. & Harland, R. M. *Early development of Xenopus laevis: a laboratory manual*. (Cold Spring Harbor Laboratory Press, 2000).

[28] Sible JC, Anderson JA, Lewellyn AL, Maller JL (1997). Zygotic transcription is required to block a maternal program of apoptosis in Xenopus embryos. Dev Biol.

