# Supplementary material for: Both Nuclear Size and DNA Amount Contribute to Midblastula Transition Timing in Xenopus laevis
Source: Sci Rep. 2017 Aug 11;7:7908. doi: 10.1038/s41598-017-08243-z (PMC5554259; doi:10.1038/s41598-017-08243-z)

SUPPLEMENTARY INFORMATION

Both Nuclear Size and DNA Amount Contribute to Midblastula Transition Timing in *Xenopus laevis*

Predrag Jevtić and Daniel L. Levy

SUPPLEMENTARY FIGURE LEGENDS

Supplementary Figure 1. GS17 in situ hybridization in embryos with altered nuclear size and ploidy

See Figure 2 legend for experimental approach.

(a) GS17 staining intensity was quantified in both halves of 7 hpf diploid and haploid control embryos, and normalized to the diploid control. N = number of embryos. Error bars represent SD. * $p < 0.05$.

(b) GS17 staining intensity was quantified in pre-MBT diploid and haploid embryo halves with increased nuclear size, and normalized to the diploid with increased nuclear size. N = number of embryos. Error bars represent SD. * $p < 0.05$.

(c) Haploid embryos were microinjected with mRNA to increase nuclear size. Cells in 4.5 hpf embryos that received the microinjected mRNA were identified by rhodamine dextran fluorescence, and nuclear size was quantified for GS17-positive and GS17-negative cells. The bottom panels show representative images of two cells that received the microinjected mRNA and showed differential GS17 staining and nuclear sizes. N = number of cells. Error bars represent SD. *** $p <0.001$. Scale bar, 20 μm.

(d) Diploid and haploid embryos were microinjected with mRNA to increase nuclear size. Cells in 4.5 hpf embryos that were positive for GS17 staining and exhibited similar

nuclear sizes were analyzed. The top graph shows average nuclear sizes, and the bottom graph shows average GS17 staining intensity for those same cells, normalized to the diploid. N = number of cells. Error bars represent SD. * $p < 0.05$; NS = not significant.

(e) Comparisons were performed between diploid control embryos and haploid embryos microinjected with mRNA to increase nuclear size. Cells in 7 hpf embryos that were positive for GS17 staining and exhibited similar nuclear sizes were analyzed. The graph on the left shows average nuclear sizes, and the graph on the right shows average GS17 staining intensity for those same cells, normalized to the diploid control. N = number of cells. Error bars represent SD. * $p < 0.05$; NS = not significant.

Supplementary Figure 2. Cell cycle timing in embryos with altered nuclear size and ploidy

See Figure 4 legend for experimental approach. Raw data used to generate Figure 4B are plotted as bar graphs with each bar representing the cell cycle length of an individual cell.

Supplemental Figure 1

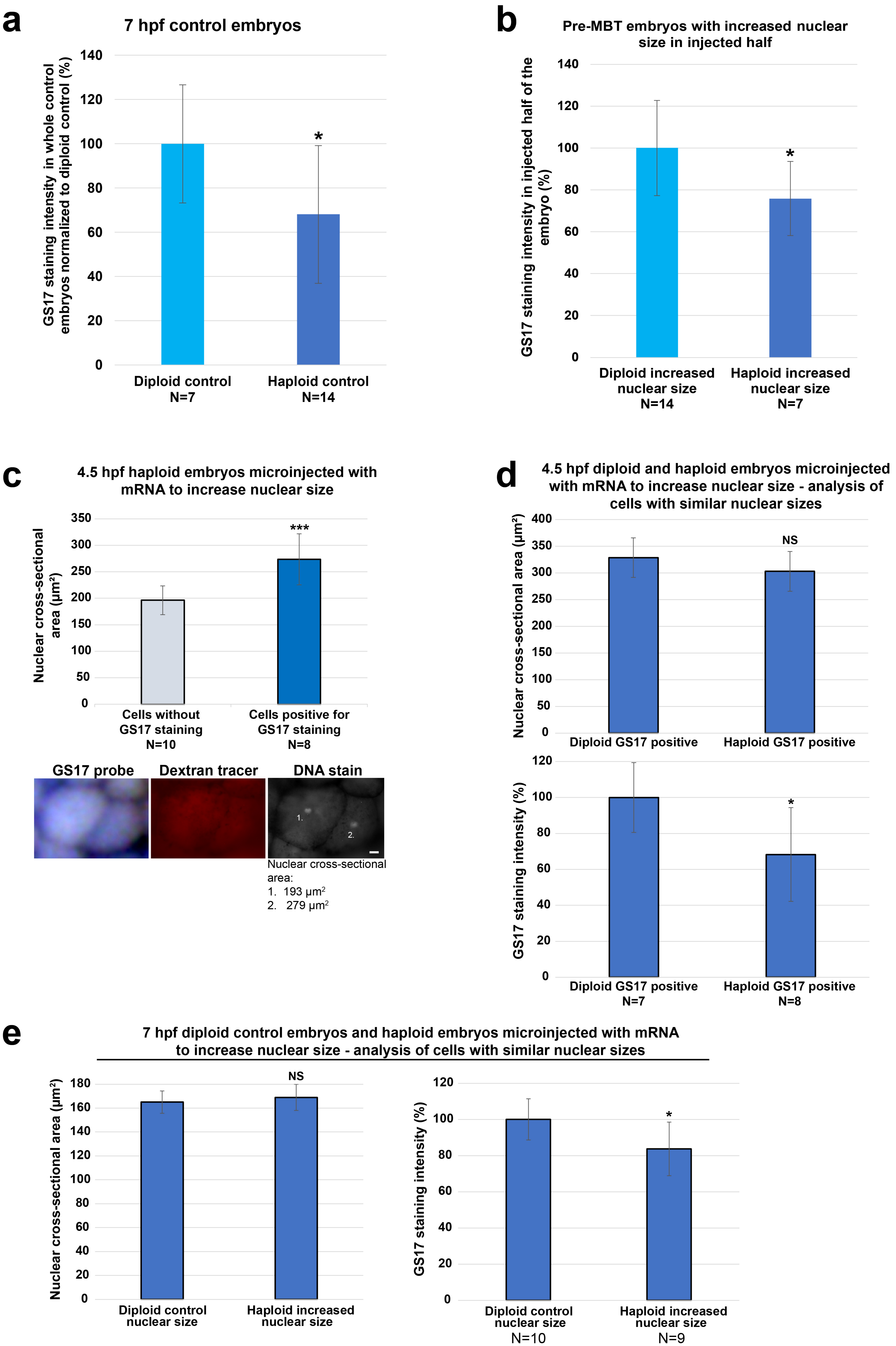

Supplemental Figure 2

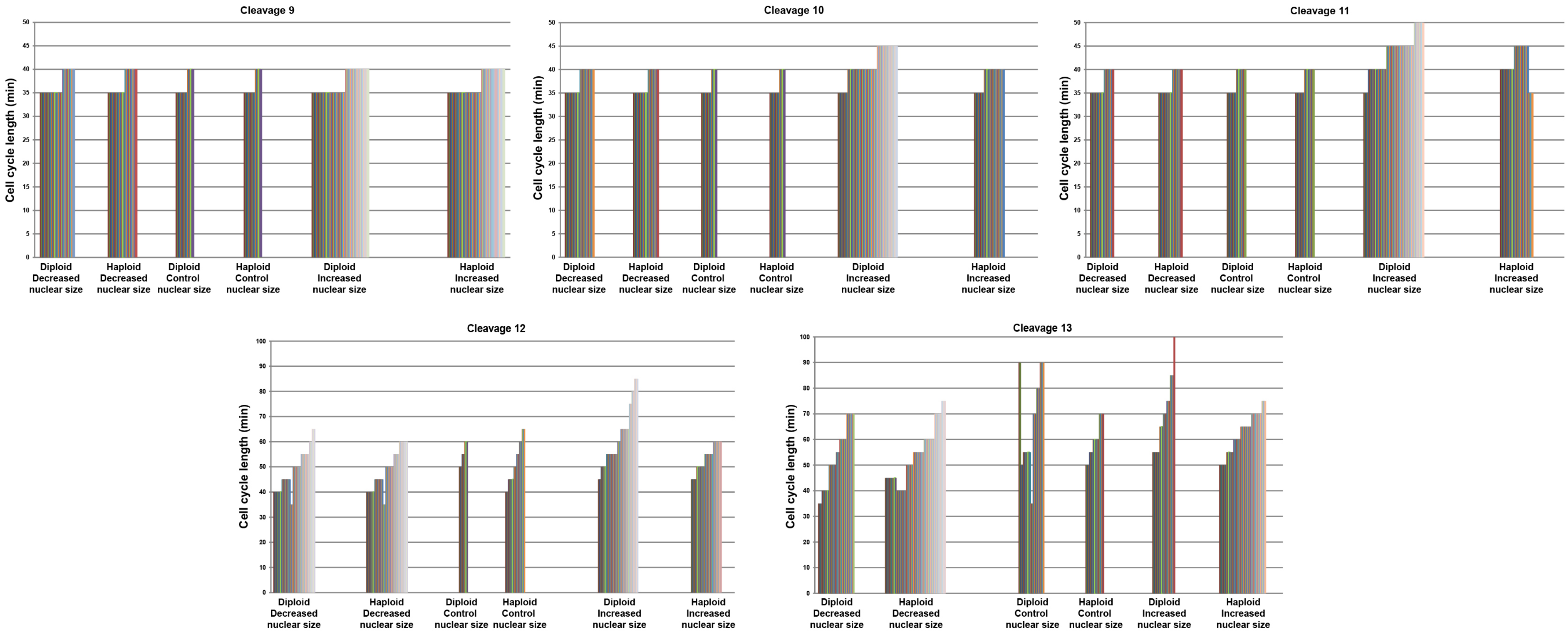

Supplement: Supplementary file 1 — Supplementary Information [file 41598_2017_8243_MOESM1_ESM.pdf]
